# Progressive Locomotor Ataxia

**Published:** 1868-11

**Authors:** Walter Coles


### Editorial Department.

### Progressive Locomotor Ataxia---By Walter Coles, M. D.

This very interesting and instuctive paper we cannot notice in detail, but copy the symptoms of the disease as given by the author:

"What, then, are the symptoms of progressive locomotor atxia? Clinical observations teach us, that this malady has no fixed mode of advent. In the majority of cases, the first approach of the disease is in the form of shooting pains, darting like electric shocks along certain portions of the limbs, more particularly the lower. These pains recur at intervals of several weeks or months, generally increasing in frequency and severity for many months or years, Sometimes they linger in the neighborhood of a joint, arousing a suspicion of rheumatism.

Though often shifting from point to point, and even from limb to limb, they chiefly confine themselves to certain nervous channels. They are oftentimes confined exclusively to one limb for a length of time before attacking the other; then, again, the lower limbs are affected simultaneously. At this juncture, or after a variable length of time, some impairment of the functions, either of the eye or its appendages, frequently becomes manifest—the patient finds himself suddenly seized with ptosis, strabismus, amblyopia, diplopia, or even triplopia of one or both eyes. In case of diplopia, objects are generally seen one above the other. The state of the pupil may be normal, contracted, or dilated. In some of these cases conjunctival congestion and chemosis are observed. Amaurosis, when once established, generally remains to the last, the other symptoms, especially those connected with the appendages of the eye, are less stable, being liable to frequent fluctuations, and sometimes disappear altogether.

Occasionally the first symptom noticed, is a sense of tingling and numbness, either in the toes or fingers, gradually spreading up the limbs or confined to special portions of it. When the hand is affected, the numbness oftener attacks the ring and little fingers, or the ulnar side of the forearm. These sensations, however, oftener come on simultaneously with the pains or after a longer or shorter interval, but almost always appear in the same limbs attacked with pain. About this time the patient complains of a feeling, as if a pad or india rubber were interposed between the soles of the feet and ground, and a peculiar unsteadiness of gait and sense of insecurity in standing. He frequently stumbles, and is liable to fall in running. These difficulties of locomotion gradually grow worse, and soon walking becomes difficult, and even impossible, without the aid of a staff or some object to lean upon. The limbs, although possessed of their normal strength, are uncontrolable by the will; they fly wildly about; the gait becomes reeling and staggering; in stepping, the foot is lifted up in a peculiar manner—is thrust forward with a spasmodic jerk, and planted forcibly, the heel coming down first. In the dark, or on closure of the eyes, these symptoms are greatly aggravated, the limbs refuse to support the body, and the patient falls to the ground, unless supported by a wall or some other object. Accompanying these symptoms is generally a marked impairment of tactile sensibility, together with the sense of muscular contraction and that of tickling. The sense of pain and differences of temperature frequently remain intact until the last. There are, however, sometimes subjective sensations of cold in the limbs. Instead of anæsthesia, there is sometimes hyperæsthesia during the earlier stages of the complaint.

In most cases the sexual powers are weakened very early in the disease; in other instances they remain in full vigor, until during the latter stages, when impotency almost always occurs. In still another class of cases, the sexual organs are unduly excited and maintain an increased vigor during a considerable length of time, (during which period nocturnal emissions are frequent,) to be finally replaced by partial or complete impotency.

The bladder, especially in the males, is nearly always affected in the progressive locomotor ataxia. At first urination is considerably more frequent. This state is sooner or later succeeded by dribbling, nocturnal incontinence, and, in some instances, paralysis of the organ. The rectum is frequently, but not so markedly affected. Besides the third and sixth, we find occasional disturbances in the fifth and seventh cranial nerves."

